# Electrical and Optical Properties of Au-Catalyzed GaAs Nanowires Grown on Si (111) Substrate by Molecular Beam Epitaxy

**DOI:** 10.1186/s11671-017-2063-3

**Published:** 2017-04-21

**Authors:** Chiu-Yen Wang, Yu-Chen Hong, Zong-Jie Ko, Ya-Wen Su, Jin-Hua Huang

**Affiliations:** 1 Department of Materials Science and Engineering, National Taiwan University of Science and Technology, Taipei, 10607 Taiwan; 2 Department of Materials Science and Engineering, National Tsing Hua University, Hsinchu, 30013 Taiwan; 3 National Nano Device Laboratories, Hsinchu, 30078 Taiwan

**Keywords:** Nanowire, Molecular beam epitaxy (MBE), GaAs, Vapor-liquid-solid (VLS)

## Abstract

In this study, defect-free zinc blende GaAs nanowires on Si (111) by molecular beam epitaxy (MBE) growth are systematically studied through Au-assisted vapor-liquid-solid (VLS) method. The morphology, density, and crystal structure of GaAs nanowires were investigated as a function of substrate temperature, growth time, and As/Ga flux ratio during MBE growth, as well as the thickness, annealing time, and annealing temperature of Au film using scanning electron microscopy (SEM), transmission electron microscopy (TEM), X-ray diffraction (XRD), cathodoluminescence (CL), and Raman spectroscopy. When the As/Ga flux ratio is fixed at 25 and the growth temperature at 540 °C, the GaAs nanowires exhibit a defect-free zinc blende structure with uniform and straight morphology. According to the characteristics of GaAs nanowires grown under varied conditions, a growth mechanism for defect-free zinc blende GaAs nanowires via Au-assisted vapor-liquid-solid (VLS) method is proposed. Finally, doping by Si and Be of nanowires is investigated. The results of doping lead to GaAs nanowires processing n-type and p-type semiconductor properties and reduced electrical resistivity. This study of defect-free zinc blende GaAs nanowire growth should be of assistance in further growth and applications studies of complex III-V group nanostructures.

## Background

Semiconductors are expected to shrink in scale to sub 10 nm. In this regard, the semiconductor industry is being severely challenged to produce semiconductor materials of suitable mobility (boosted processing speed) and architecture (for reduced power leakage) on a nanometric scale [1]. There is an urgent need for the development of semiconductor materials that can address this problem. Since unique electrical and optical properties are required, III-V group semiconductors have been proposed as candidates to replace Si as a high-speed device material [2–5]. One such material is gallium arsenide (GaAs). It is representative of III-V group semiconductor materials and possesses a direct bandgap of 1.424 eV [6, 7]. III-V group semiconductor nanowire arrays of high volume to surface ratios are potential materials whose bandgap can be tuned for the efficient transfer of solar energy to electric energy [8–10]. The literature presents various examples of the growth of binary and ternary III-V group semiconductor nanowires with controlled bandgaps on various substrates. Among these studies, single-crystal GaAs is used as a homogeneous substrate for growing GaAs semiconductor nanowires [11, 12]. However, single-crystal GaAs substrate is expensive and difficult to integrate into the present Si-based industry. On this basis, Si substrates are more desirable in support of III-V group semiconductor nanowire growth via vapor-liquid-solid (VLS) mechanism or deposit of a GaAs film as a buffer layer [13–15]. VLS as a growth method is popular as it facilitates reduced growth temperatures. Lower growth temperatures inhibit strain and defects causing lattice mismatching. Furthermore, small lattice mismatch also helps zinc blende GaAs to overcome strain issue as grown on Si substrate. Si is diamond structure and its lattice constant is 0.5431 nm, on the other hand, GaAs is zinc blende structure and its lattice constant is 0.5653 nm. The small lattice mismatch, ~4%, leads to GaAs nanowires could epitaxial growth on Si substrate [15]. There are many studies point that diameters, density, and quality of nanowires/nanorods are influenced by growth parameters, such as thickness and annealing temperature via Au-catalyzed VLS growth mechanism [16–19]. Over the past several years, the synthesis of GaAs nanowires (NWs) on Si has predominantly been investigated using metal organic vapor phase epitaxy via a Au-catalyzed VLS mechanism [20–24]. On the other hand, few studies have employed molecular beam epitaxy (MBE) technique. In this systematic study, GaAs nanowires are grown under an MBE system on Si (111) via Au-catalyzed VLS mechanism. The investigation includes thickness and annealing conditions of the Au film, Ga to As flux ratio, growth temperature, dopant (Si and Bi) effects on electrical properties, and optical properties.

## Methods

Samples were grown on Au film-coated Si (111) substrates in a Varian Modular GEN-II MBE system. Before using an e-gun to deposit thickness-varied Au film (0.6, 1, 1.5, and 2.0 nm), the silicon native oxide on the Si (111) substrate is removed by 1 wt% HF then rinsed with DI water and dried with N_2_ gas. In this work, all MBE system sources for growing nanowires are solid elements (including Ga, Be, Si, and As) and their flux is controlled using a temperature controller and shutter under 1 × 10^−10^ Torr. Au film is annealed to form nanoparticles on the Si (111) substrate then Ga and As are provided to grow GaAs nanowires. For in situ Si- and Be-doped GaAs nanowires, the concentrations of Si and Be are tuned by controlling the solid elements temperature. Doping temperatures of Si and Be are 1250~1400 °C and 1000~1150 °C, respectively. The corresponding concentrations and carrier species of Si- and Be-doped GaAs samples are calibrated by Hall measurement to confirm. To prepare samples for Hall measurement, (111) Si substrate without Au film is used to deposit GaAs films with different dopant source temperatures. SEM images were obtained using a HITACHI-S4700 field-emission SEM, operated at 5–15 kV accelerating voltage. The TEM samples were prepared by drop-casting nanostructures from toluene dilute dispersions onto 200-mesh carbon-coated copper grids (Electron Microscope Sciences). Energy dispersive spectrometry was conducted using a 200-kV accelerating voltage on a JEOL JEM-2100F. X-ray diffraction (XRD) was performed with a Rigaku Ultima IV X-ray diffractometer using Cu Kα radiation (λ = 1.54 Å) with of 1°/min scan rate. A cathodoluminescence (CL) detector was attached to SEM, and Raman spectrum was measured through Horiba, HR 800 by 633 nm laser and electrical properties measured by Keithly-590 for I-V curves.

## Results and Discussion

**Fig. 1:**
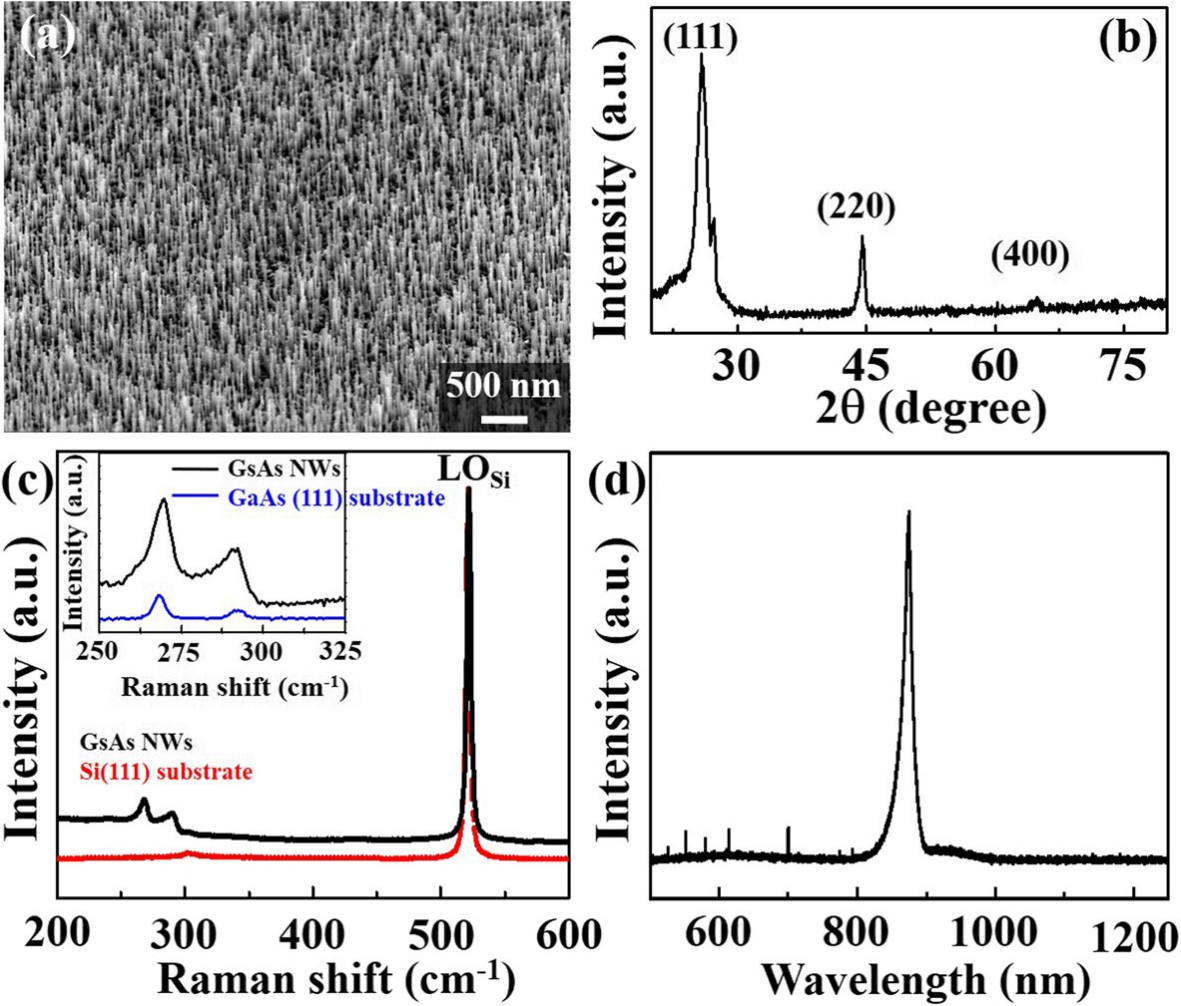
SEM, XRD, Raman and CL. **a** The SEM image of GaAs nanowires grown. **b** The products were analyzed by grazing incident X-ray diffraction (XRD). **c** Raman spectra and the peaks at 291 and 267 cm^−1^ contribute from GaAs zinc blende structure. The *inset* is as-grown GaAs NWs and zinc blende structure of (111) GaAs wafer. **d** CL spectrum result that Au-catalyzed growth GaAs nanowires excited by 15 kV electron beam in SEM at room temperature

The growth temperature window of Au-catalyzed GaAs nanowires is 520–620 °C [4, 8]. In this work, the optimal conditions for Au film thickness and annealing temperature are 1.0 nm and 580 °C for 10 min, respectively. Figure 1a shows a representative SEM image of Au-catalyzed GaAs nanowires grown on Si (111) substrate at 540 °C for 15 min with Ga/As flux ratio of 1/25. The diameter and length of Au-catalyzed GaAs nanowires are 26.2 ± 3.4 nm and 424.4 ± 13.4 nm, respectively. The products were further analyzed using grazing-incident X-ray diffraction (XRD). The result shown in Fig. 1b indicates that GaAs nanowires are crystalline and all peaks can be ascribed to a zinc blende structure with lattice constants of *a* = 0.5653 nm (JCPDS No. 89-2770). A strong (111) peak implies that the GaAs nanowires are textured on the Si (111) substrate. Since arsenic evaporates from GaAs at high temperatures, a high-As ambience is needed to produce high-quality GaAs nanowires. The growth rate of Au-catalyzed GaAs nanowires is limited by the participation of Ga from saturated AuGa_*x*_ alloy. Environmental Ga atoms react with excess As to form polycrystalline GaAs on Si (111) surface, and the taper-shaped GaAs nanowires will be formed [14]. Raman spectroscopy is shown in Fig. 1c. Peaks at 291 and 267 cm^−1^ are contributed by GaAs zinc blende structure. No wurtzite structure peaks are found at 259 cm^−1^ (the peak at 520 cm^−1^ is contributed from the Si substrate). The bandgap of the zinc blende structure and wurtzite structure of GaAs are 1.424 and 1.46 eV at 300 K, respectively; their corresponding illumination wavelengths are 870 and 850 nm. Figure 1d is the CL result of Au-catalyzed growth GaAs nanowires that measured at room temperature. The density of GaAs NWs and thickness of lateral grown GaAs layer are different for varied growth conditions that the CL intensity of GaAs NWs does not be compared but their full width at half maximum (FWHM) is similar. Furthermore, the CL peak at 870 nm confirms the product of GaAs nanowires is a zinc blende structure. The CL results show that the strong emission signal at 870 nm relates to the bandgap of the GaAs array nanowires. Because the CL peak is sharp, the Au-catalyzed growth GaAs array nanowires must have a good crystalline structure. No other peak was detected by CL spectrometry meaning no other defect was introduced into Au-catalyzed growth GaAs array nanowires. Even if high-concentration dopants of Si or Be are added into the Au-catalyzed growth GaAs array nanowires, the spectra of Raman and CL are consistent with XRD results in Fig. 1b.

**Fig. 2:**
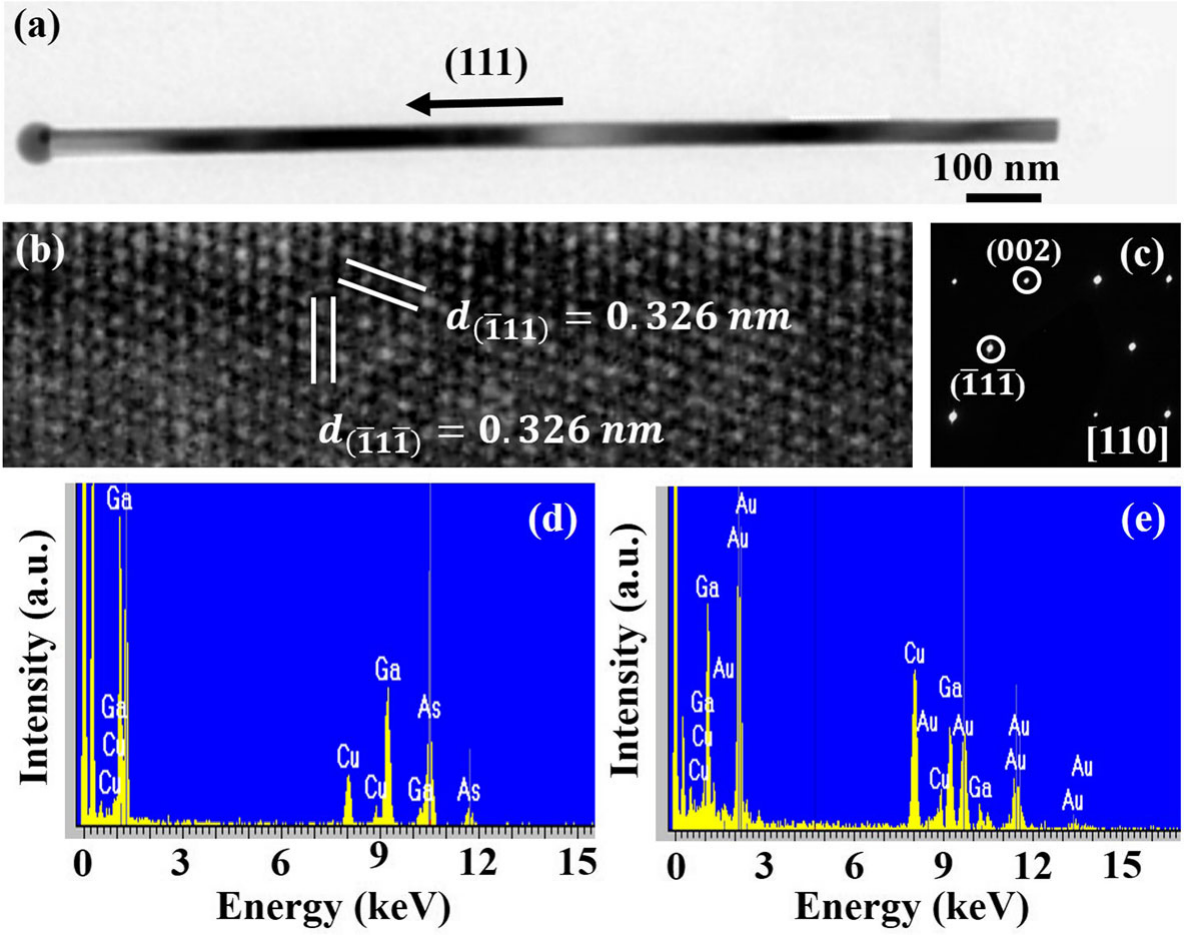
TEM, HRTEM, SEAD, and EDS. **a** A TEM image of a representative Au-catalyzed GaAs nanowire. The corresponding high-resolution TEM (HRTEM) image and SAED pattern are shown in (**b**) and (**c**), respectively. **d**, **e** The EDS spectra of the corresponding NW that taken from top of AuGa_*x*_ and stem, respectively

Figure 2a gives a bright-field transmission electron microscope (TEM) image of a representative Au-catalyzed GaAs nanowire. An AuGa_*x*_ alloy nanoparticle with dark contrast is clearly shown at the top of the straight and smooth GaAs nanowire. A high-resolution TEM (HRTEM) image and selected area electron diffraction (SAED) pattern of the corresponding GaAs nanowire are shown in Fig. 2b, c, respectively. The HRTEM image shows that the Au-catalyzed GaAs nanowire has a single-crystalline structure and (111) planes are observable along the [110] direction with a 3.26-nm spacing. The SAED pattern result in Fig. 2c further confirms the Au-catalyzed GaAs nanowire is a zinc blende structure grown in the (111) direction. This result is consistent with XRD, Raman, and CL results in Fig. 1b–d. Figure 2d, e is the energy dispersive spectrometer (EDS) spectra of Au-catalyzed GaAs nanowires taken from the top of the AuGa_*x*_ nanoparticle and stem of the GaAs nanowire, respectively. EDS spectrum of Fig. 2d shows the compositions of the nanoparticle at the top are Au and Ga without As proving that the GaAs nanowires are catalyzed by AuGa_*x*_ droplets and As atoms react with Ga segregated at AuGa_*x*_-GaAs interface. The ratio of Ga/As of EDS spectrum in Fig. 2e is about 1 meaning Au-catalyzed GaAs nanowires are grown at a moderate growth rate. The signal peaks for Cu and C come from the carbon-coated copper grid used as the electron transmission holder to suspend GaAs nanowires for TEM analysis.

**Fig. 3:**
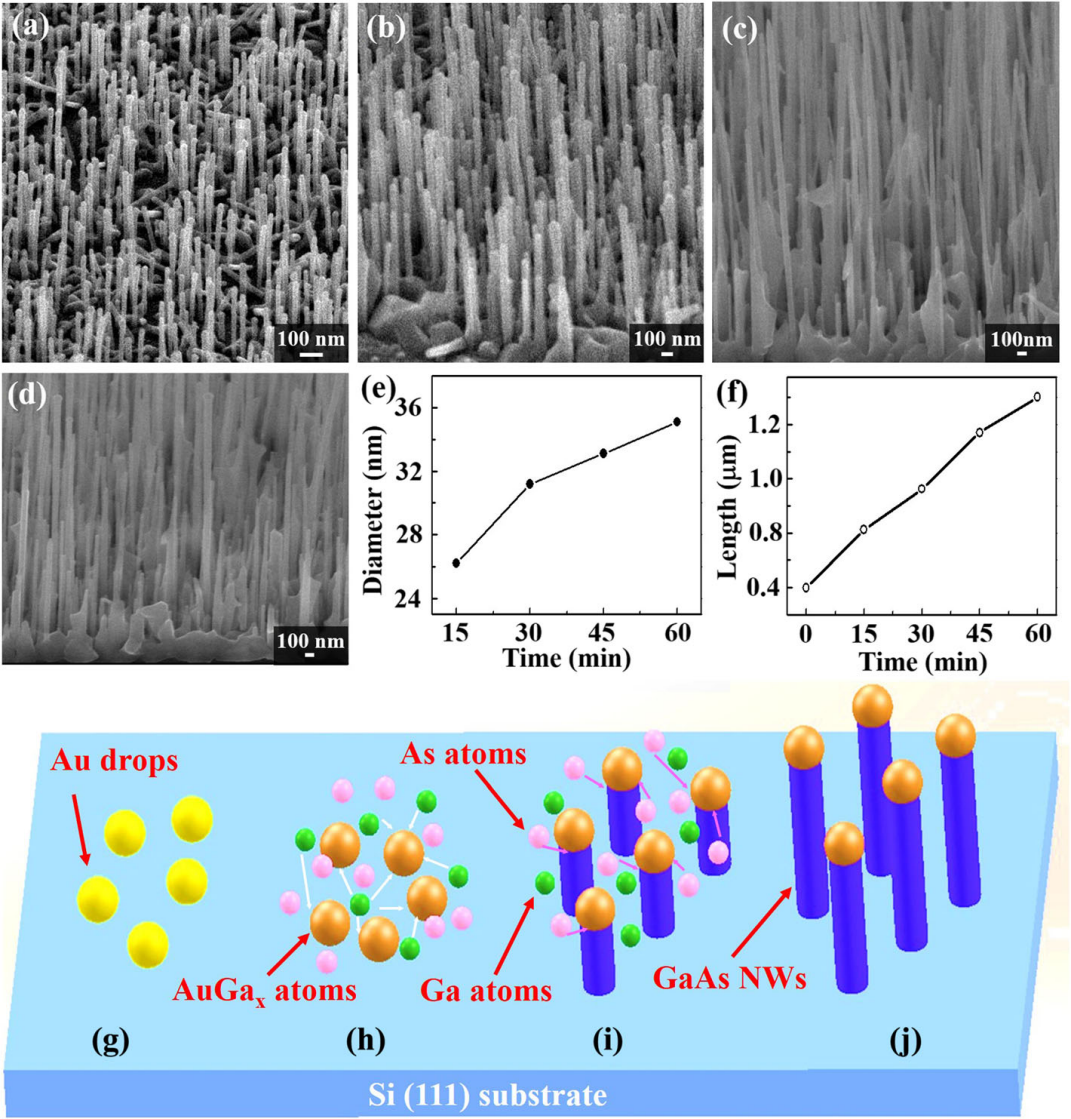
SEM, diameter-time, length-time, and growth mechanism. SEM images show the length and diameter of GaAs NWs with varied growth time, for (**a**) 15, (**b**) 30, (**c**) 45, and (**d**) 60 min. **e, f** Varied growth time vs the relationship of diameter and growth length, respectively. **g**–**j** The schematics illustrating the growth of Au-catalyzed GaAs NWs : **g** the Au film annealed to form Au drops, **h** Au drops alloy with Ga to produce the AuGa_*x*_ alloy catalyst, and **i** the segregation of Ga out of the AuGa_*x*_ catalyst can be achieved as the AuGa_*x*_ alloy catalyst reaches a supersaturation condition. **j** Form the axial growth of GaAs NWs

**Fig. 4:**
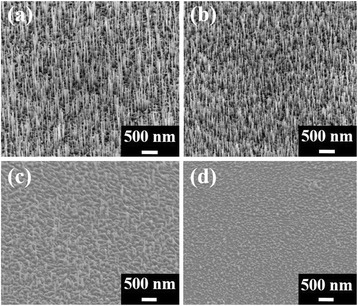
**a**-**d** are SEM images of 0.6, 1.0, 1.5 and 2.0 nm Au film deposited by e-gun, respectively, then annealed at 580 °C for 10 min to study the influence of Au thickness in GaAs NWs growth. Ga flux is 1.0×10^-7^ Torr and As flux is 2.5×10^-6^ Torr and grown at 540 °C for 15 min

As growth times increase (15, 30, 45, and 60 min), the length and diameter of GaAs nanowires increases but so too does the surface roughness. Longer GaAs nanowires are formed when As atoms experience longer diffusion paths to reach Au-Ga interface to form GaAs. Further, environmental Ga reacts with As at the sidewall of GaAs nanowires increasing nanowire diameters. SEM images in Fig. 3a–d show the length and diameter of GaAs nanowires with time: (a) 15, (b) 30, (c) 45, and (d) 60 min. The length of GaAs nanowires grows from hundreds of nanometers to micrometers, and diameters increase from 26.2 to 35.1 nm. These results indicate the importance of controlling Ga and As flux to manage GaAs nanowire morphology and surface roughness. Figure 3e, f shows growth time vs nanowire diameter and length, respectively. The results imply that the growth rate is not limited by diffusion since the length of GaAs nanowires is linearly proportional to the growth time not the root mean square of time (*t* ^1/2^) [25, 26]. It is likely controlled by precipitation of Ga from the interface of liquid AuGa_*x*_ droplets and solid GaAs nanowires or the As diffusion rate. Tapered structures are observed in Fig. 3c, d as growth time increases [14]. As mentioned above, this is caused by As experiencing a longer diffusion path before reacting with Ga at the interface between liquid AuGa_*x*_ droplets and solid GaAs, as well as As atoms reacting with precipitated ambient Ga atoms provided by the MBE chamber to produce GaAs at the sidewall of nanowires forming tapered structures. Since tapering and surface roughness increase with time and these factors may negatively affect optimal optical and electrical properties of GaAs nanowires, we analyze the optical and electrical properties of nanowires grown at a fixed time of 10 min. Figure 3g–j give Au film thickness, As flux, annealing temperature, and growth temperature for Au-catalyzed GaAs nanowires. The figures show the progression of a typical VLS nanowire growth mechanism. Initially, Au film annealed to form droplets (Fig. 3g) then an Au-Ga alloy forms to produce the AuGa_*x*_ alloy catalyst (Fig. 3h). Subsequently, Ga is segregated from the AuGa_*x*_ under supersaturation conditions (Fig. 3j) to form axial growth of GaAs nanowires. From SEM images, the fast growth rate of Au-catalyzed GaAs nanowires suppresses lateral growth meaning overcoating on the surface of GaAs nanowires does not occur. The exclusive supply of Ga and As atoms from AuGa_*x*_ droplets results in this fast growth rate with no lateral overgrowths. Fewer lateral overgrowths means fewer nanowire defects. Varied Au films, 0.6, 1.0, 1.5, and 2.0 nm, deposited by e-gun then annealed at 580 °C for 10 min are used to study the influence of Au thickness in GaAs NW growth. SEM images in Fig. 4 display Au film cannot form drop as its thickness is thicker than 1.5 nm; on the other hand, thinner Au film, 0.6 nm, leads to grow lower density of GaAs NWs.

**Fig. 5:**
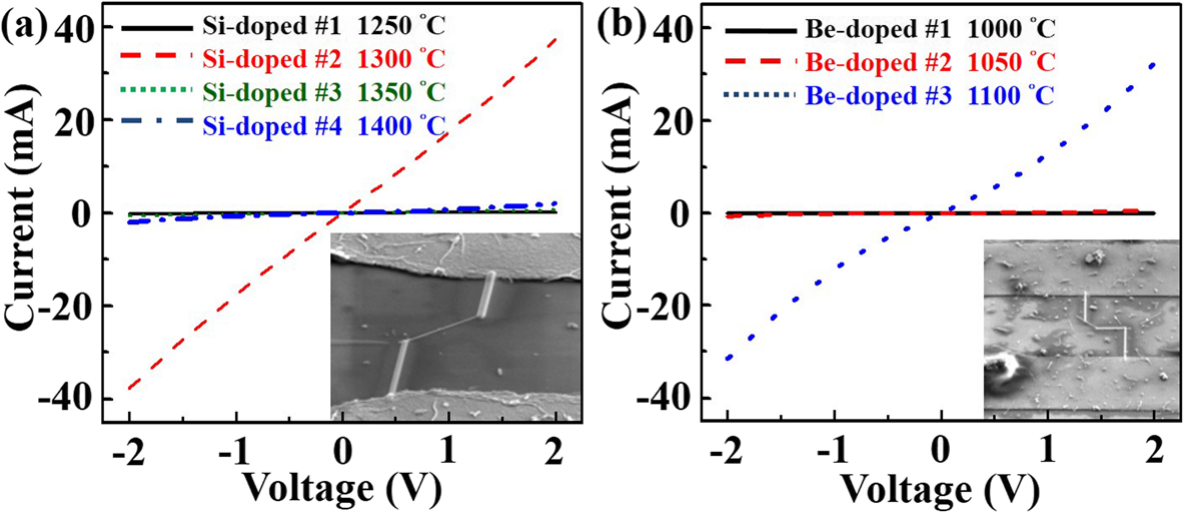
Current-voltage measurements of SI-doped and Be-doped nanowires. **a**, **b** Electrical measurements with different dopants concentration Si and Be, respectively

In situ Si and Be doping effects are investigated in terms of the electrical properties of Au-catalyzed GaAs nanowires with different dopant source temperatures to control doping concentrations. In this work, FIB is used for depositing Pt as contact electrodes for the electrical characterization. Figure 5f, g is the results of electrical measurements with different concentrations of Si and Be dopants, respectively. The I-V curves of Fig. 5a are Si dopant source temperatures at 1250, 1300, 1350, and 1400 °C for fabricating single Au-catalyzed GaAs nanowire devices for measurement. High Si source temperatures mean high concentrations of Si; however, I-V measurements show lower conductivity for Si source at higher than 1300 °C. According to XRD, TEM, CL, and Raman results of GaAs grown at 1350 °C, the structure is still zinc blend. Since Si is an amphoteric dopant for GaAs, it seems that As sites are substituted by partial Si atoms. Be is a typical n-type dopant for a GaAs donor. In this work, Be source temperatures are 1000, 1050, and 1100 °C for adjusting Be concentrations in the Au-catalyzed GaAs nanowires. Figure 5b shows the corresponding I-V measurement results of varied Be source temperatures. As temperature increases, conductivity increases meaning donor Be atoms successfully substitute As sites enhancing carrier transport. Furthermore, the dopant concentrations affect to the electrical resistivity but do not change the structure of GaAs nanowire as the doping levels are 10^19^~10^18^/cm^3^ and 10^15^~10^18^/cm^3^ with Si and Be as a dopant, respectively. Concentrations of Si and Be doped GaAs samples are calibrated by Hall measurement to confirm whether the carrier types are electrons or holes. To prepare samples for Hall measurement, (111) Si substrate without Au film is used to deposit GaAs films with different dopant source temperatures. Table 1 gives the results of Si-doped and Be-doped Au-catalyzed GaAs nanowire electrical measurements. Electrical resistivity is determined by single Au-catalyzed GaAs nanowire devices, carrier concentrations, and carrier species are determined by Hall measurement.

**Table 1:** The results of Si-doped and Be-doped Au-catalyzed GaAs nanowires electrical measurements, including electrical resistivity that determined by single Au-catalyzed GaAs nanowire devices, reference layer doping concentration, and carrier species are determined by Hall measurements

Sample	Resistivity (Ω m)	Reference layer doping (cm^−3^)	Carrier species
Si-doped 1250 °C	7.89 × 10^−2^	2.75 × 10^19^	Electron
Si-doped 1300 °C	5.98 × 10^−2^	3.95 × 10^19^	Electron
Si-doped 1350 °C	2.69 × 10^−3^	8.92 × 10^19^	Electron
Si-doped 1400 °C	4.31 × 10^−1^	2.51 × 10^18^	Electron
Be-doped 1000 °C	4.30 × 10^−3^	1.12 × 10^18^	Hole
Be-doped 1150 °C	4.39 × 10^−2^	5.92 × 10^16^	Hole

## Conclusions

Au-catalyzed GaAs nanowires were grown using an MBE system. Diameter and length density were controlled for thickness, annealing time, annealing temperature of the Au film, Ga/As ratio, and growth temperature. A growth model for Au-catalyzed GaAs nanowires was elucidated based on the analysis of SEM, XRD, and TEM results. Electrical properties of Au-catalyzed GaAs nanowires can be adjusted by controlling concentrations of Be and Si dopants through source temperatures. The carrier species and concentrations in Au-catalyzed GaAs nanowires are calibrated through Hall measurement at room temperature. High Si dopant source temperatures affect substitution sites; further, different concentrations of Si-doped and Be-doped Au-catalyzed GaAs nanowires are fabricated as single devices to measure their electrical resistivity and compare these results using Hall measurement.

## Abbreviations

MBE: Molecular beam epitaxy
VLS: Vapor-liquid-solid
SEM: Scanning electron microscopy
TEM: Transmission electron microscopy
XRD: X-ray diffraction
CL: Cathodoluminescence
HRTEM: High-resolution TEM
SAED: Selected area electron diffraction
EDS: Energy dispersive spectrometer
